# Supplementary material for: The interactome of intact mitochondria by cross-linking mass spectrometry provides evidence for coexisting respiratory supercomplexes
Source: Mol Cell Proteomics. 2017 Dec 8;17(2):216–32. doi: 10.1074/mcp.RA117.000470 (PMC5795388; doi:10.1074/mcp.RA117.000470)
Supplement: Supplemental Data [file supp_RA117.000470_133922_0_supp_23978_fzffwf.zip › spectra_annotation/mito_DR_spectra_annotation/484-1-7-1-5-1.pdf]

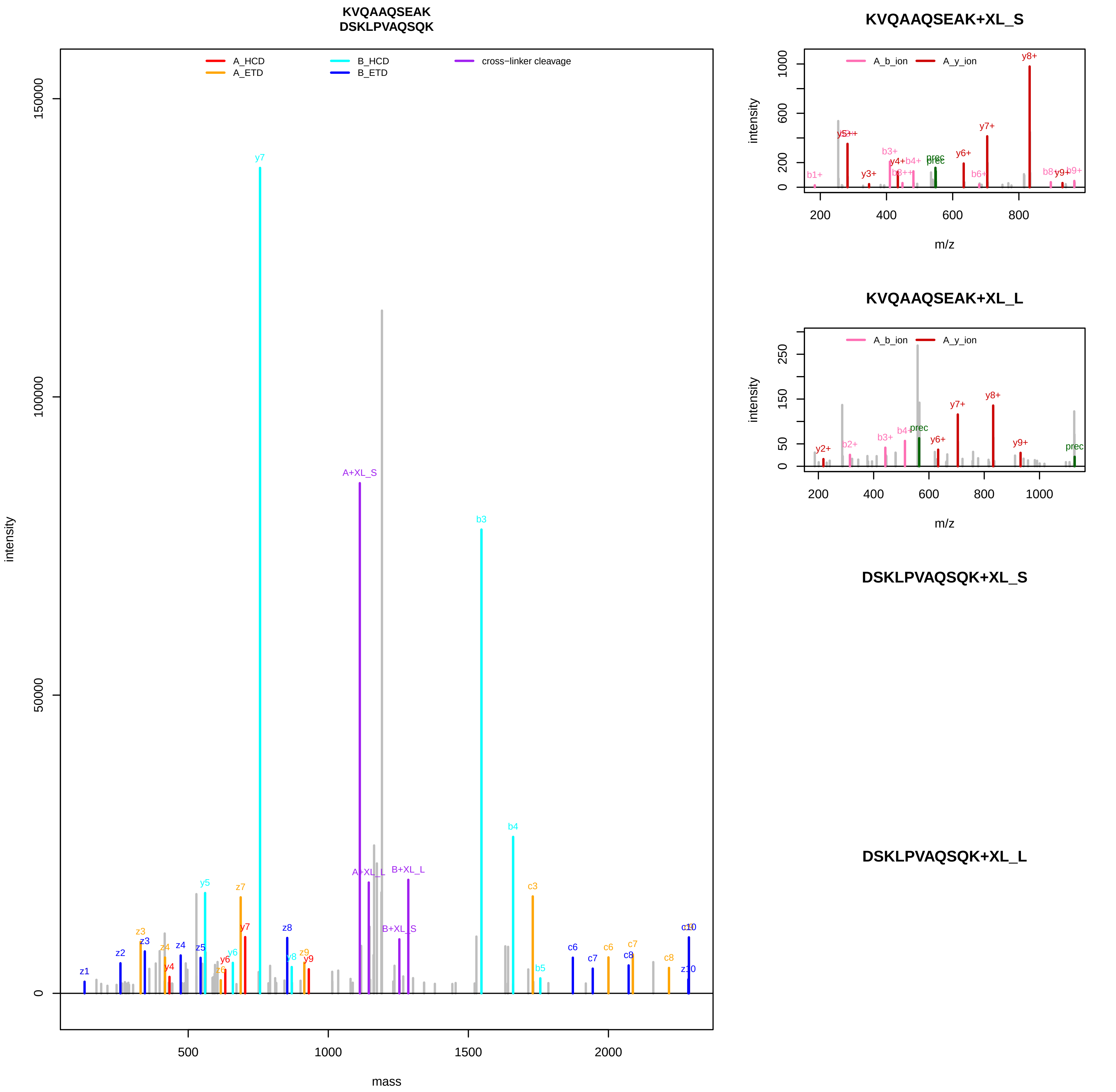
KVQAAQSEAK
DSKLPVAQSQK
A_HCD
A_ETD
B_HCD
B_ETD
cross−linker cleavage
intensity
mass
0
50000
100000
150000
500
1000
1500
2000
y7
A+XL_S
b3
b4
c3
A+XL_L
B+XL_L
B+XL_S
y5
z7
z8
z3
z2
z1
z4
z5
y6
y4
y7
y9
z9
y8
b5
c6
c7
c8
c10
z10
KVQAAQSEAK+XL_S
A_b_ion
A_y_ion
m/z
y8+
y7+
y6+
b3+
y4+
b4+
prec
y3+
b1+
b6+
b8+
y9+
b9+
KVQAAQSEAK+XL_L
y2+
b2+
y8+
y7+
y6+
y9+
DSKLPVAQSQK+XL_S
DSKLPVAQSQK+XL_L
